# Genome-Wide Transcriptional Changes and Lipid Profile Modifications Induced by *Medicago truncatula* N5 Overexpression at an Early Stage of the Symbiotic Interaction with *Sinorhizobium meliloti*

**DOI:** 10.3390/genes8120396

**Published:** 2017-12-19

**Authors:** Chiara Santi, Barbara Molesini, Flavia Guzzo, Youry Pii, Nicola Vitulo, Tiziana Pandolfini

**Affiliations:** 1 Department of Biotechnology, University of Verona, 37134 Verona, Italy; chiara.santi@univr.it (C.S.); barbara.molesini@univr.it (B.M.); flavia.guzzo@univr.it (F.G.); nicola.vitulo@univr.it (N.V.); 2 Faculty of Science and Technology, Free University of Bozen-Bolzano, 39100 Bolzano BZ, Italy; youry.pii@unibz.it

**Keywords:** lipid transfer proteins, root nodule symbiosis, transcriptomic analysis, lipid profile

## Abstract

Plant lipid-transfer proteins (LTPs) are small basic secreted proteins, which are characterized by lipid-binding capacity and are putatively involved in lipid trafficking. LTPs play a role in several biological processes, including the root nodule symbiosis. In this regard, the *Medicago truncatula* nodulin 5 (MtN5) LTP has been proved to positively regulate the nodulation capacity, controlling rhizobial infection and nodule primordia invasion. To better define the lipid transfer protein MtN5 function during the symbiosis, we produced MtN5-downregulated and -overexpressing plants, and we analysed the transcriptomic changes occurring in the roots at an early stage of *Sinorhizobium meliloti* infection. We also carried out the lipid profile analysis of wild type (WT) and MtN5-overexpressing roots after rhizobia infection. The downregulation of MtN5 increased the root hair curling, an early event of rhizobia infection, and concomitantly induced changes in the expression of defence-related genes. On the other hand, MtN5 overexpression favoured the invasion of the nodules by rhizobia and determined in the roots the modulation of genes that are involved in lipid transport and metabolism as well as an increased content of lipids, especially galactolipids that characterize the symbiosome membranes. Our findings suggest the potential participation of LTPs in the synthesis and rearrangement of membranes occurring during the formation of the infection threads and the symbiosome membrane.

## 1. Introduction

Plant lipid transfer proteins (LTPs) constitute a large family of small proteins (6.5–10.5 kDa), with an isoelectric point commonly ranging from 8.5 to 12, characterized by the capacity to bind and transport hydrophobic molecules [1,2,3]. Plant LTPs are often reported as non-specific LTPs (nsLTPs), because of their capacity to associate with various phospholipids with broad specificity [4]. All of the identified nsLTPs contain a conserved 8 cysteine motif and an N-terminal signal peptide for the targeting of the mature form toward the secretory pathway [5]. 

Non-specific LTPs show variable expressions in different tissues and developmental stages, and under diverse biotic and abiotic stresses. For instance, members of the nsLTP family are regulated by drought, temperature, wounding, as well as phytohormones, such as abscisic acid and methyl jasmonate [6]. These features are indicative of a great variety of biological functions that are played in plants [7,8,9]. Various biological roles have been proposed for plant LTPs, including defence against pathogenic microorganisms and insect pests [10,11,12,13,14], formation of cuticle, and interaction between pollen and stigma [14]. LTPs have been demonstrated to play a role also during the symbiotic association between leguminous plants and nitrogen-fixing bacteria [15,16,17].

The relationship between leguminous plants and nitrogen fixing bacteria leads to the formation of a new organ, the root nodule, where the bacteria eventually fix nitrogen. Nodule formation represents the end-point of a complex developmental program, which involves the infection of the epidermal cells and concomitantly the activation of cortical cells divisions. The formation of a functional nodule needs a spatial and temporal coordination between these two events [18]. The symbiotic dialogue is triggered by the plant, which releases into the soil signalling molecules, including flavonoids, which are sensed by rhizobia. In response, rhizobia synthesize the Nodulation Factors (NFs), molecules consisting of a lipochitooligosaccharide (LCO) backbone of *N*-acetylglucosamine subunits decorated by a variety of substituents, which determines the specificity of interactions between different rhizobia and host legume species [18,19]. The perception of NFs by host-plant, as mediated by LysM receptor-like kinases (LysM-RKs), triggers a downstream signal transduction cascade, including cellular and developmental responses, such as the modification of ion fluxes, Ca^2+^ oscillations, membrane depolarization, cytoskeletal reorganization, root hair deformations, and the activation of cell division [18,19]. Arbuscular mycorrhizal fungi also produce LCO molecules, namely the Myc factors, which are structurally similar to NFs [19]. The perception of both NFs and Myc factors induces in the root the so-called common symbiosis (SYM) pathway, which is the signalling pathway that prepares the plant for the successful infection with both rhizobia and mycorrhizal fungi [18,19]. Besides the SYM pathway, other independent signalling pathways are required for the initiation of the infection. Following signal exchanges, rhizobia enter root hair via a tubular plant-derived structure, named infection thread (IT), and then they are eventually released into the cortical layers, where the bacteria enclosed in a plant-derived membrane called symbiosome, enter redifferentiated cells of the nodule [20,21,22,23,24]. Inside the nodule, bacteroids can undergo to diverse differentiation processes, depending on the legume clade to which the host plant belongs [25]. In legumes belonging to the inverted repeat-lacking sequence clade (IRLC), such as *Medicago*, rhizobia are usually subjected to endoreduplication, cell elongation, and terminal differentiation, coupled to the loss of the ability to replicate outside the host root [26]. In almost all the legumes belonging to IRLC, a key role in the terminal differentiation of bacteria into nitrogen-fixing bacteroids is played by nodule-specific cysteine-rich (NCR) peptides. Some NCRs are similar to defensins and possess antimicrobial activity in vitro [27,28,29,30]. During bacteroid differentiation, the symbiosome membrane undergoes lipid rearrangements and modifications of transport proteins [31]. The targeting of new lipids and protein material is under the control of both host plant and bacteria [32]. In this regard, in Chinese milk vetch (*Astragalus sinicus*), a winter growing green manure legume, which can establish a symbiotic interaction with *Mesorhizobium huakuii*, the nodule-specific lipid transfer protein AsE246 participates in the transport of plant-synthesised lipids to the symbiosome membrane and is essential for nodule organogenesis [17].

In a different symbiotic relationship (i.e., *Medicago truncatula*/*Sinorhizobum meliloti*) another nsLTP, the nodulin 5 (N5), was shown to control the host response to the rhizobia [15,16]. The lipid transfer protein MtN5 was classified as an early nodulin, and is thus recognized as a precocious marker of the symbiosis [33]. *MtN5* expression is induced after rhizobial infection in the epidermis and root hairs and at a later stage in nodule primordia and nodules [15,16]. By generating transgenic roots via *Agrobacterium rhizogenes* transformation, MtN5 was demonstrated to be required for the successful establishment of the symbiotic interaction because *MtN5* silencing determined a reduced number of invaded primordia and nodules, whereas *MtN5* overexpression resulted in an increased nodulation capacity [15,16]. MtN5 acts upstream of Flotillin 4 (FLOT4), a protein that was localised into membrane microdomains of root hair cells and required for proper formation and growth of IT [34], and independently from Does not make infection 1 (*DMI1*), a component of the SYM pathway [16,35,36]. The lipid binding capacity of MtN5 suggests a role either in the remodelling of membranes during rhizobial infection or in the signalling between rhizobia and host cells [36]. However, a comprehensive knowledge about the precise role of MtN5 during the early symbiotic events remains to be elucidated.

The aim of this work was to deeper explore the function of MtN5 by analysing the transcriptomic changes produced in *M. truncatula* roots by the down regulation and overexpression of *MtN5* at an early stage of *S. meliloti* infection. We also investigated the changes in lipid profile induced in rhizobia-infected roots by the overexpression of *MtN5*. On the basis of these analyses, we identified the principal pathways that are associated with MtN5 function, and revealed that MtN5 participates in the lipid remodelling induced by rhizobia infection.

## 2. Materials and Methods

### 2.1. Microbial Strains

*Sinorhizobium meliloti* 1021 was grown at 28 °C in LBMC medium (10 g/L tryptone, 5 g/L yeast extract, 171 mM NaCl, 2.6 mM MgSO_4_, 2.6 mM CaCl_2_) supplemented with streptomycin 200 μg/mL [37]. *S. meliloti* 1021/pXLGD4 vector containing the *hemA::lacZ* reporter was grown at 28 °C in LBMC medium supplemented with streptomycin 100 μg/mL and tetracycline 10 μg/mL [38]. *Rhizophagus irregularis* DAOM 197198 DAOM 181602 spores were purchased from Belgian Co-ordinated Collections of Microorganisms/Mycothèque de l’Université catholique de Louvain, Belgium (BCCM/MUCL).

### 2.2. Medicago truncatula Stable Transformation with MtN5-Silencing and MtN5-Overexpression Constructs

The gene constructs for *MtN5* silencing (MtN5hp) and *MtN5* overexpression (MtN5ox) were built following the constructs design previously described [15], using the constitutive 35S promoter from Cauliflower mosaic virus as promoter regulatory sequence. For MtN5hp construct, the arms of the hairpin construct consist of two 200 bp-long fragments from base +93 to +292 of the N5 coding sequence (Y15371) placed in inverted orientation and separated by the spliceosomal intron of the *MtLAX* gene. For MtN5ox construct, the sequence coding for the mature form of MtN5 was used. The genetic constructs were cloned into a derivative of the pBIN19 vector and the resulting recombinant plasmids were moved into *Agrobacterium tumefaciens* cells (strain GV2260) [39]. For *Medicago* transformation and in vitro plant regeneration, we used the protocol reported in [40].

### 2.3. Plant Growth and Symbiont Inoculation

*Medicago truncatula* cv. Jemalong (J5) seeds were scarified and sterilized in 5% commercial bleach for 3 min and germinated on plant agar 0.8%. Plants used for rhizobium inoculation were grown in a mixture 1:1 of sterile sand and perlite, supplemented once a week with 10 mL of a solution of micro- and macro-elements (0.13 mM KH_2_PO_4_, 0.3 mM CaCl_2_, 0.06 mM MgSO_4_, 0.2 mM K_2_SO_4_, 0.014 mM FeNa-EDTA, 1.56 μM H_3_BO_3_, 1.24 μM MnSO_4_, 4.5 μM KCl, 0.11 μM ZnSO_4_, 0.1 μM CuSO_4_, 0.32 μM H_2_SO_4_, and 2.1 μM Na_2_MoO_4_). Four-week-old plantlets were transferred from sand-perlite pots to hydroponic culture containing BNM medium (2 mM CaSO_4_, 0.5 mM KH_2_PO_4_, 0.5 mM MgSO_4_, 2 mM MES pH 6.5, 50 μM EDTA, 50 μM FeSO_4_, 16 μM ZnSO_4_, 50 μM H_3_BO_3_, 50 μM MnSO_4_, 1 μM Na_2_MoO_4_, 0.1 μM CuSO_4_, 0.1 μM CoCl_2_) and *S. meliloti* culture at optical density (O.D.)_600_ = 0.1 was added. Wild type (WT) plants were mock-inoculated with a solution of 10 mM MgSO_4_. To obtain *M. truncatula* plants colonized by the AM fungus *R. irregularis*, one-week old plantlets were transferred into sterile sand containing the fungal spores and were supplemented once a week with 10 mL of Hoagland half-strength solution depleted of phosphate salt (11 μM H_3_BO_3_, 2.6 μM MnSO_4_, 3.4 μM ZnSO_4_, 1.9 μM CuSO_4_, 1 μM FeNa EDTA, 18 mM Ca(NO_3_)_2_, 15 mM KNO_3_, 10 mM MgSO_4_, 2 mM NaCl). Wild type were mock-inoculated with the Hoagland half-strength solution. All of the plants were grown in a growth chamber at 25 °C, with a regimen of 10 h of light and 14 h of darkness at 120 µmol m^−2^ s^−1^.

### 2.4. LacZ Histochemical Staining

Plants inoculated with *S. meliloti* (final O.D._600_ = 0.1) bearing the *hemA::LacZ* reported gene were stained, as described in [36]. Whole root samples were mounted on glass slides with coverslips and observed with a Leica DM2500 microscope equipped with a DFC420C digital camera (Leica Microsystems, Wetzlar, Germany).

### 2.5. Cutin Treatment

For cutin treatments, seven-day-old *M. truncatula* seedlings were placed on Petri dishes containing slanted Fåhraeus Modified agar Medium (1 mM CaCl_2_, 0.5 mM MgSO_4_, 0.7 mM KH_2_PO_4_, 0.8 mM Na_2_HPO_4_, 0.5 mM NH_4_NO_3_, 50 μM FeNaEDTA, 0.66 μM MnSO_4_, 0.63 μM CuSO_4_, 0.62 μM ZnSO_4_, 1.62 μM H_3_BO_3_, and 0.48 μM Na_2_MoO_4_) supplemented with 16-hydroxypalmitic acid at two concentrations (20 µg/mL and 50 µg/mL) and roots sampled after 48 h.

### 2.6. Trypan Blue Staining

Mycorrhized plants were stained following the protocol described in [41] at 15, 45, and 60 days post inoculation (dpi). Roots were cleared in hot 10% KOH, acidified with 2% HCl, and stained in 0.05% trypan blue that was dissolved in lactic acid. Root samples were then mounted on glass slides with coverslips and observed with a Leica DM2500 microscope equipped with a DFC420C digital camera (Leica Microsystems).

### 2.7. RNA Extraction and Quantitative RT-PCR

Total RNA was isolated using the NucleoSpin^®^ RNA plant kit (Macherey-Nagel, Düren, Germany) and quantified using the NanoDrop™ 1000 spectrophotometer (Thermo Scientific, Schwerte, Germany). RNA quality was evaluated using Agilent 2100 Bioanalyzer (Agilent, Waldbronn, Germany). Three complementary DNA (cDNA) samples derived from three independent RNA samples were analysed. cDNA amplification and reverse transcription PCR (RT-PCR) cycling conditions and product dissociation curve were carried out, as indicated in [42]. Data from quantitative Reverse Transcription-PCR (qRT-PCR) experiments were analysed according to the 2^−ΔΔCt^ method [43]. The list of primers adopted for qRT-PCR analyses is reported in Supplementary Table S4.

### 2.8. Microarray and Data Analyses

Three independent pools of WT, *MtN5*-overexpressing and *MtN5*-silenced roots from plants inoculated for 72 h with *S. meliloti* were sampled. Complementary RNAs (cRNAs) were synthesized starting from 200 ng of total RNA isolated, as above described and labelled with Cyanine 3 (Cy3)-CTP fluorescent dye, according to the manufacturer’s instructions (Agilent). Then 1.65 μg of Cy3-labeled cRNA of each sample was used for subsequent hybridization to the *M. truncatula* oligo microarray with the design number 022524. After hybridization for 17 h at 65 °C, slides were washed and scanned with an Agilent Microarray Scanner (G2565CA, Agilent). Scanned images were transformed into quantified figures by the Agilent features extraction software, and the expression data were normalized based on the 75th percentile value. Statistical analysis (*t*-test, *p*-value < 0.01) was carried out using T-MeV v4.9.0 software (http://mev.tm4.org) to define significantly modified genes between MtN5ox and WT roots and MtN5hp and WT roots. Fold changes were calculated as the ratio between the averages of the three replicates of samples compared. Differentially expressed transcripts were filtered based on the fold change values (FC ≥ |1.5|).

### 2.9. Probe Mapping to M. truncatula Genes and Annotation

The *M. truncatula* reference genome and the gene prediction gff3 file (version Mt4.02v2) were downloaded from *M. truncatula* Genome Database website (http://www.medicagogenome.org). The microarray probes were aligned on the reference genome using hisat2 program [44], and all of the probes mapping on multiple location were removed. Finally, we performed the association between the probes and the *M. truncatula* genes using the ‘intersect’ module implemented in bedtools package (http://bedtools.readthedocs.io/), which reports overlaps between two feature files. Bedtools program was run providing the gff3 gene prediction file and the probe alignment bam file. *M. truncatula* gene annotation, GO terms and InterPro annotations were retrieved from Biomart platform at the EnsemblPlants web site (https://plants.ensembl.org/biomart/martview/).

### 2.10. Lipid Extraction

The plants (WT, MtN5hp and MtN5ox) were grown for four weeks in sand and perlite mixture, and were then inoculated with *S. meliloti.* After 72 h, freshly excided roots were washed in distilled water; for each sample three independent pools, each one containing the whole root apparatus from 10 individual plants, were assembled and stored at −80 °C. The lipidic fraction of the metabolome was extracted from the frozen powder, obtained by grinding the root in liquid nitrogen with an IKA mill model A11 (IKA^®^ Works, Wilmington, DE, USA). Ten volumes of 2/1 chloroform/methanol (*v*/*v*) and 200 µL of deionized water were added to about 180 mg of frozen powder. Samples were accurately mixed, sonicated at 40 kHz in an ultrasonic bath (Falc Instruments, Bergamo, Italy) for 15 min, centrifuged at 10,600× *g* for 5 min at 4 °C. The chloroform phase was collected, vacuum dried, and resuspended in three volumes (initial weight) of methanol and filtered. 

### 2.11. LC-MS, Metabolite Annotation and Data Analysis

Lipid HPLC-MS (High Performance Liquid Chromatography-Mass Spectrometry) analysis was performed with a Beckman Coulter Gold 127 HPLC system (Beckman Coulter, München, Germany) coupled online with a Bruker ion mass spectrometer Esquire 6000 (Bruker Daltonik GmbH, Bremen, Germany) equipped with an APCI (Atmospheric Pressure Chemical Ionization) ionization source. Chromatographic elution was carried out with an analytical Alltima HP C18 column (150 × 2.1 mm, particle size 3 µm) equipped with a C18 guard column (7.5 × 2.1 mm) (Alltech Associates Inc., Columbia, MD, USA), by using a mixture of solvent A (0.5% (*v*/*v*) formic acid, 5% (*v*/*v*) acetonitrile in LC-MS-grade water), and solvent B (100% acetonitrile). Starting from 50% of solvent B, a gradient was established from 50% to 100% solvent B in 10 min, followed by isocratic elution at 100% acetonitrile for 65 min and return to initial conditions in 1 min. The column was equilibrated for 14 min in 50% solvent B. Negative and positive ion mass spectra were recorded in the range 50–1500 *m*/*z* (full scan mode, 13,000 *m*/*z* s^−1^). Nitrogen was used as the nebulizing gas (50 p.s.i.) and drying gas (10 L/min). Helium was used as the collision gas. The other parameters were set as follows: trap drive, 61.3; octopole radio frequency amplitude, 200 Vpp; lens −60 V; Capillary exit, 143.5 V; dry temperature, 350 °C; APCI temperature, 450 °C; high voltage (HV) capillary, 4000 V; HV end-plate offset, –500 V. Since parent ion were mostly detectable in the negative ionization chromatograms, most of lipid annotation, performed using the fragmentation trees, were carried on negative MS/MS and MS3 spectra, recorded in the range 50–1500 *m*/*z*, with the fragmentation amplitude set at 1 V. DGDG (DiGalactosyl Diacyl Glycerol) and MGDG (MonoGalactosyl Diacyl Glycerol) were annotated, as previously described [45], in negative ionization mode, while GlcCer (Glucosyl Ceramides) were annotated, as described in [46], in the positive ionization mode; Phosphatidyl Ethanolamine (PE) containing phospholipids were annotated based on neutral loss of 141, corresponding to PE, in positive ionization mode. MS data were collected and processed with on Esquire Control v5.2-Esquire-Data Analysis v3.2 (Bruker Daltonik GmbH) and MZmine v. 2.21 (http:// mzmine.sourceforge.net), and the data were imported into a Microsoft Excel 2010 (Microsoft, Redmond, WA, USA) data sheet for analysis with SIMCA-P +v12.0 (Umetrics AB, Umeå, Sweden). All of the variables (Intensity of signal of lipids, in arbitrary units) were centred and scaled to Pareto Variance. The data matrix was analysed by PCA (Principal Component Analysis).

## 3. Results

### 3.1. Transcriptional Analysis of MtN5-Silenced and MtN5–Overexpressing Plants at an Early Stage of S. meliloti Infection

**Figure 1:**
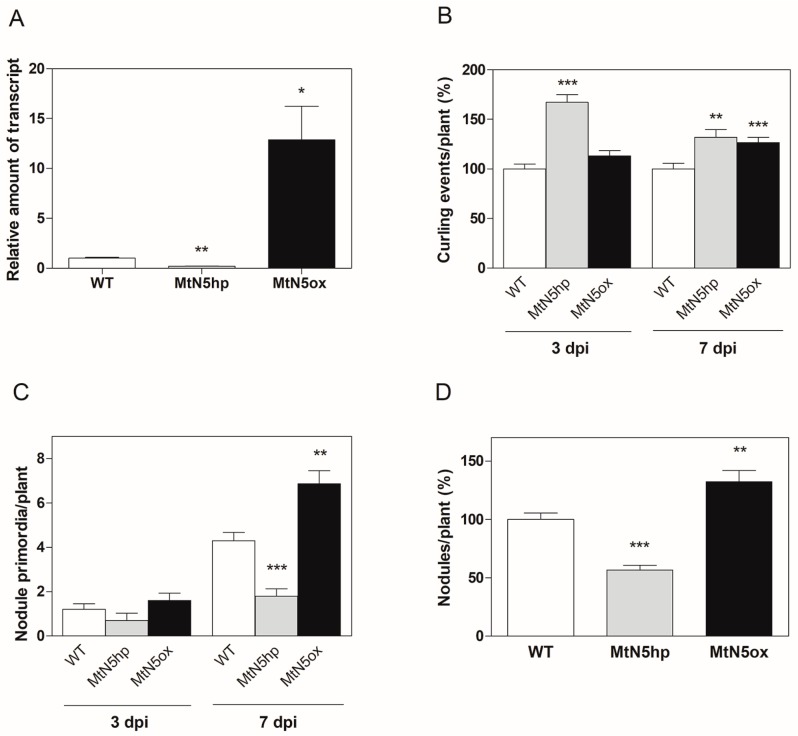
Lipid transfer protein *MtN5* gene-silencing and overexpression in *Medicago truncatula* plants. (**A**) Quantitative Reverse Transcription-PCR (qRT-PCR) analysis of *MtN5* mRNA level in leaves of wild type (WT) and stably transformed with the silencing vector MtN5hp and the overexpression vector MtN5ox into plants. The data reported are means ± standard error (SE) (*n* = 3). (**B**) Number of root hair curling events per plant and (**C**) number of invaded primordia per plant in MtN5hp and MtN5ox roots inoculated with *Sinorhizobium meliloti* at 3 days post inoculation (dpi) and at 7 dpi. The data reported in (**B**) was calculated as percentage relatively to WT. The data reported are means ± SE (*n* = 10). (**D**) Number of mature nodules per plant in WT, MtN5ox and MtN5hp roots. The data reported are means ± SE (*n* = 20) calculated as percentage relatively to WT inoculated roots. Student’s *t*-test was applied. * *p* < 0.05; ** *p* < 0.01; *** *p* < 0.001.

Previous studies carried out on transgenic roots obtained via *A. rhizogenes* revealed that the *MtN5* downregulation impairs nodulation, whereas its overexpression increases root nodulation capacity [15]. To further investigate the role of *MtN5* in the symbiotic interaction with *S. meliloti*, we performed a genome-wide transcriptomic analysis of roots of *M. truncatula* plants transformed via *A. tumefaciens* harbouring either a silencing (MtN5hp) or an overexpressing construct (MtN5ox). The MtN5hp and MtN5ox plants, showing a ~80% reduction and 10 times increase in *MtN5* transcript level, respectively (Figure 1A), were phenotypically characterised. 

Stable transformed MtN5hp and MtN5ox plants did not revealed any pronounced visible morphological change as compared with WT plants, neither in the roots nor in the shoots (data not shown). This suggests that the principal role of MtN5 is related to the host response to the rhizobia.

To assess whether the response of stable transformed plants to rhizobia infection was similar to what was observed in roots of composite plants [16], we performed inoculation experiments with a rhizobium strain harbouring the *hemA::LacZ* reporter gene [38,47]. We examined the effects of the rhizobium infection after three and seven dpi. In MtN5hp, the number of root hair curling events was significantly increased in comparison to WT plants at both three and seven dpi (Figure 1B). In MtN5ox, an increased number in root hair curling was observed at seven dpi (Figure 1B). Albeit, root hair curling was enhanced in both MtN5hp and MtN5ox roots, the number of invaded primordia displayed an opposite trend (Figure 1C). MtN5hp showed a reduced number of invaded primordia as compared with control plants, whereas in MtN5ox, the number of invaded primordia increased (Figure 1C). MtN5hp and MtN5ox plants showed ~40% decrease and ~30% increase in the number of mature nodules as compared with WT plants, respectively (Figure 1D). These data confirm that MtN5 acts as a positive regulator of the nodulation process (this work and [16]). The increased number of curling events caused by MtN5 downregulation might represent a feed-back control over the root hair infection as observed also in other *M. truncatula* mutants impaired in nodulation [36].

**Figure 2:**
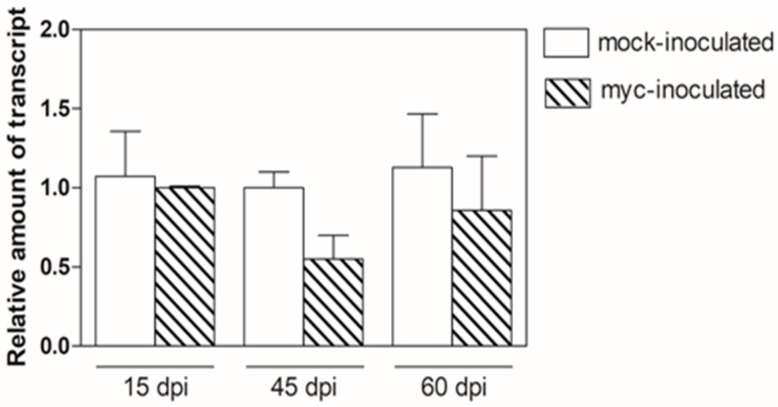
qRT-PCR analysis of *MtN5* mRNA level in mycorrhized roots at 15, 45, and 60 dpi calculated versus mock-inoculated roots. Values reported are means ± SE (*n* = 3).

When considering that rhizobia and arbuscular mycorrhizal fungi produce signalling molecules that can activate a common SYM signalling pathway, we assessed the effects of mycorrhizal infection on *MtN5* expression. The expression level of *MtN5* was assayed in inoculated WT roots at 15, 45, and 60 days post infection with *R. irregularis*. At all of the times tested, *MtN5* expression was unaffected (Figure 2).

In addition, we did not detect differences in the capacity of *R. irregularis* to colonize WT, MtN5hp, and MtN5ox roots (Supplemental Figure S1A). We also tested the response of *MtN*5 to cutin monomers that act as signalling molecules during the first phases of the mycorrhizal symbiosis [48]. *MtN5* expression was evaluated in WT roots after 24 h of treatment with 16-hydroxypalmitic acid, which is a major component of cutin. The steady state level of *MtN5* mRNA did not vary after cutin treatment (Supplementary Figure S1B).

Global gene expression of WT MtN5hp and MtN5ox roots was analysed at 72 h post inoculation with *S. meliloti*, a time that under our experimental conditions corresponds to symbiotic events ascribable to the initial phase of the infection (i.e., many adhesion zones and curling events, rare nodule primordia) [41]. Three independent biological replicates per sample were used and each biological replicate consisted of root apparatuses from eight plants. Genes were considered differentially expressed if the fold change (FC) was ≥|1.5| and the *p* value (Student’s *t-*test) was *p* < 0.01. The pairwise comparison between rhizobia-inoculated MtN5hp and WT roots resulted in 74 differentially expressed genes (DEGs), 52 upregulated, and 22 downregulated (Supplementary Table S1). In the roots of MtN5ox plants that were inoculated with *S. meliloti,* 939 genes were differentially expressed, 427 upregulated, and 512 downregulated (Supplementary Table S2). The two data sets showed an overlap of only eight transcripts. 

**Figure 3:**
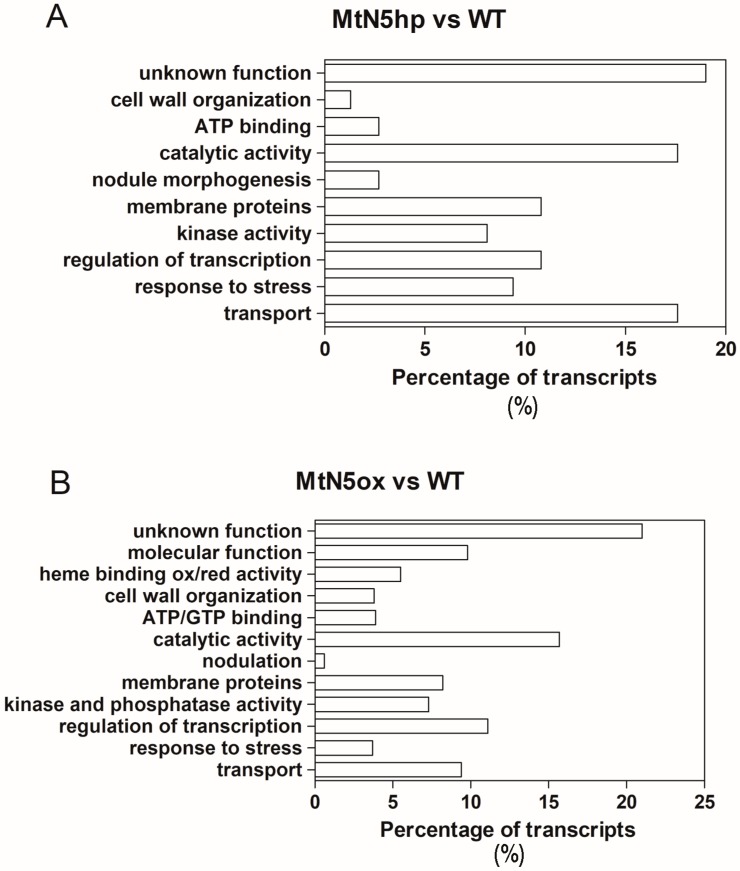
Main functional categories of differentially expressed genes (DEGs). Differentially regulated transcripts in the comparison MtN5hp vs. WT (**A**) and MtN5ox vs. WT (**B**) were grouped into main functional categories. Transcripts without any annotation information were collected into ‘unknown function’ category.

To assign the annotations to the differentially expressed genes, the microarray probes were aligned on the Mt4.02v2 version of the *M. truncatula* genome sequence (http://www.medicagogenome.org). After delating the probes that match multiple locations in the genome, the remaining probes were associated to *M. truncatula* genes, as described in Material and Methods (Section 2.9). The DEGs were grouped into functional categories based on either their assigned molecular function or biological process Gene Ontology (GO) terms (Figure 3A,B).

The expression level of four randomly chosen genes was quantified by qRT-PCR, and were found to be consistent with the microarray data (Supplementary Figure S2).

### 3.2. Changes in Gene Expression Observed in MtN5hp Inoculated Roots

It was possible to assign a function to ~81% of the DEGs detected in the comparison MtN5hp vs. WT (Figure 3A and Supplementary Table S1). Among the annotated DEGs, those that were related to transport were well represented (Figure 3A). A member of the major intrinsic protein (MIP) transporter family (*Medtr8g098375*) was downregulated. Major intrinsic proteins are abundant in the symbiosome membrane. These proteins generally act as aquaporins, but in the symbiosome can also facilitate the movement of other solutes, such as NH_4_ [49]. Other DEGs that are involved in ion transmembrane transport include proteins belonging to the DUF (Domain of Unknown Function) family, a heavy metal transporting P-type ATPase (*Medtr4g127580*), and a chloride channel (*Medtr4g134730*). The last one is of particular note because chloride, together with calcium and potassium, is rapidly mobilized in root hairs in response to NFs [50]. Nodulation Factors cause an increase in the cytosolic free Ca^2+^, that in turn triggers Cl^−^ efflux, depolarizing the plasma membrane and alkalizing the root hair apoplastic space [50]. These events seem to participate in root hair deformation and in the subsequent entrapment and internalization of bacteria [51]. In addition, two transcripts coding for ABC-type transporters (*Medtr3g040090*, *Medtr2g102660*) were upregulated in MtN5hp roots and a triose phosphate/phosphate translocator coding transcript (*Medtr4g088350*) was downregulated.

Several genes that are involved in the response to biotic stress were modulated in MtN5hp roots (Figure 3A and Supplementary Table S1), for instance, two transcripts coding for protein involved in disease resistance (*Medtr4g021037*, *Medtr8g446950*) and a cationic peroxidase (*Medtr7g107520*) were upregulated, whereas a glutaredoxin-C1 protein (*Medtr1g088910*), an antioxidant enzyme involved in the protection against oxidative damage and biotic stress, was 78-fold reduced, as well as two transcripts coding for protein putatively involved in response to disease (*Medtr6g036500*, *Medtr7g018850*). In addition, we detected a gene (*Medtr4g081655*) coding for a S-locus receptor kinase protein whose expression was a three-fold increase in MtN5hp inoculated roots as compared with WT inoculated ones. S-locus receptor kinases (SD-RLKs), a subfamily of lectin receptor kinases, are implicated in the self-incompatibility in flowering plants, a process that can be described as an endogenous defence strategy to prevent inbreeding [52]. Due to the resemblance of SD-RLK extracellular domain with lectin proteins, known to bind to fungal and bacterial cell wall components, lectin receptor kinases are hypothesized to participate in biotic stress tolerance [53]. Recently, it has been reported that a lectin S-domain receptor kinase mediates lipopolysaccharide sensing in *Arabidopsis thaliana* [54]. 

Regarding the genes playing a role in rhizobia symbiosis (Figure 3A and Supplementary Table S1), we found two transcripts coding for NCR secreted peptides (*Medtr4g065390*, *Medtr3g027180*), which were upregulated in MtN5hp inoculated roots in comparison to WT ones with a fold change of 23 and 3.6, respectively. Nodule cysteine rich are nodule specific proteins targeted to the symbiosome, where they induce bacteroid development [30,55]. In addition, a gene coding for a γ-glutamyl hydrolase (*Medtr2g075780*), that was found upregulated in a proteomic survey carried out on a supernodulating soybean variety at two days after inoculation [56], resulted repressed in MtN5hp roots. 

The data set of MtN5hp inoculated roots showed an overlap of seven annotated DEGs with that of MtN5ox inoculated ones. Among these, DEGs coding for a chloride channel (*Medtr4g134730*), a glutaredoxin-C1 protein (*Medtr1g088910*), an UDP-D-glucose/UDP-D-galactose 4-epimerase (*Medtr5g009160*), and an auxin efflux carrier (*Metr8g107360*) showed a similar modulation trend in both MtN5hp and MtN5ox roots. The remaining DEGs coding for a ubiquitin protein ligase (*Medtr5g020570*), a calcium-dependent lipid binding protein (*Medtr7g076900*), and a sugar porter (*Medtr2g020710*) displayed an opposite behaviour. 

### 3.3. Changes in Gene Expression Observed in MtN5ox Inoculated Roots 

The 939 DEGs were grouped into functional categories (Figure 3B and Supplementary Table S2), 196 of them corresponded to genes with unknown function. We have focused our analysis on DEGs that are related to ‘regulation of transcription’ and ‘transport‘ which are highly-represented in our dataset, and on DEGs that are related to symbiotic interaction and lipid metabolism. 

#### 3.3.1. Transcription Factors

The ‘regulation of transcription’ category comprises 104 DEGs, most of them encode transcription factors (TFs) (Figure 3B and Supplementary Table S2). The no apical meristem (NAM), Arabidopsis transcription activation factor (ATAF), cup shaped cotyledon (CUC2) (NAC), myeloblastosis virus (MYB), and ethylene responsive (AP2/ERF) TFs families were the most represented. In plants, NAC proteins participate in various biological processes including root nodule symbiosis. For instance, NAC1 of M. *truncatula* was found to be induced in young and mature nodules [57]. Another member of this family, NAC969, is involved in lateral root emergence and nodule senescence [58]. Its overexpression caused an increase in lateral roots number, while its silencing provoked an anomalous accumulation of amyloplasts in the nitrogen-fixing zone of the nodules, and nodules were prematurely senescent [58]. In MtN5ox-inoculated roots, we observed the modulation in the expression of nine members of the NAC-like TF family. Nine transcripts coding for MYB TFs were downregulated in MtN5ox plants, whereas four were upregulated. The documented regulatory roles played by MYB TFs in leguminous plants are mainly related to abiotic stresses, flavonoid metabolism, and nodulation [59]. In *Glycine max*, 64 of 244 MYB transcripts were expressed during the nodulation process, and 39 of them showed the highest expression in nodule tissues [59]. A soybean MYB transcription factor, named control of nodule development (CND), participates in the regulation of nodule development [60].

Five members of basic helix-loop-helix (bHLH) TFs were downregulated in MtN5ox plants, and two were up-regulated. To date, in *M. truncatula* genome more than 100 *bHLH* genes were identified, but a few of them were proved to have a role in the nodulation process and/or in the root system development [61]. In this regard, *MtbHLH1* expression has been detected in nodule primordia and in the vascular tissue of the nodules [62]. Transgenic silenced roots *MtbHLH1* developed nodules with vascular irregularities [62]. One *bHLH* gene of *Lotus japonicus,* named Roothariless1, has been shown to be fundamental in root hair development and in root colonization by the rhizobia [63]. Another member of this family, the *Symbiotic Ammonium Transporter 1* (*SAT1*), initially documented as a putative NH_4_^+^ ammonium transporter of the symbiosome membrane, was subsequently demonstrated to be a bHLH TF, with a potential regulatory activity on a plasma membrane NH_4_^+^ channel [64]. Loss of activity of the *SAT1* in soybean roots impaired nodule development and reduced nodule numbers and fixation capacity [64]. 

Eight ethylene responsive TFs (i.e., AP2/ERF) and four GRAS TFs were modulated in the comparison MtN5ox vs. WT. In *M. truncatula,* members of the GRAS TFs, i.e., Nodulation-signalling pathway 1 and 2 (NSP1 and NSP2) and of AP2-ERF TFs, i.e., ERF-required for nodulation (ERN 1, ERN2, and ERN3), are well known regulators that are implicated in root hair infection and nodule development [65]. 

#### 3.3.2. Transport

In the comparison MtN5ox vs. WT, we detected 164 modulated transcripts belonging to the ‘transport’ and ‘membrane protein’ functional categories, 67 were upregulated and 97 downregulated (Supplemental Table S2). Most of the downregulated DEGs are implicated in the transport of organic and inorganic form of nitrogen (Table 1). 

Seven genes code for proteins involved in amino acid movement (Medtr6g016275, Medtr1g109380, Medtr8g085630, Medtr2g012470, Medtr7g017630, Medtr8g089340, Medtr4g104510), four for peptide/nitrate transporters (Medtr5g038060, Medtr4g114340, Medtr3g100980, Medtr6g008690), three for oligopeptide transporters (Medtr0294s0020, Medtr8g061090, Medtr4g098800), and one nitrate transporter (Medtr7g088820). Notably, leguminous plants engage symbiotic interaction with nitrogen-fixing bacteria only under conditions of nitrogen starvation; the presence of relative high concentration of nitrate in the medium inhibits the symbiosis, and can lead to the suppression of nodule development [66]. We also detected numerous DEGs related to the transport of other inorganic nutrients. The expression of genes coding for protein involved in the transport of iron (Medtr5g068580) and magnesium (Medtr2g028770) was induced, whereas genes that were related to the uptake of zinc (Medtr4g083570), phosphate (Medtr1g075640, Medtr1g041695, Medtr3g082700), sulphate (Medtr4g011600, Medtr1g071530, Medtr3g087740), and chloride (Medtr4g134730, Medtr6g065650) were downregulated (Table 1). In addition, four members of the MIP/aquaporin family (Medtr3g070210, Medtr1g006490, Medtr8g087710, Medtr2g094270) were downregulated, and the expression of three transcripts, coding for potassium channels (Medtr8g088200, Medtr3g108320, Medtr4g099260) were altered (Table 1). Several transcripts coding for proteins implicated in sugar transport were modulated in the comparison MtN5ox vs. WT (Table 1). The establishment of the symbiotic process requires an energy demand, and therefore alterations in source-sink relationships also via modifications of the sugar transport. Consistently, the sucrose synthase, which is responsible for the cleavage of sucrose transported from the shoots, was demonstrated to be necessary for an efficient nitrogen-fixation in several leguminous plants [67,68], but it is also induced during the first phases of the symbiotic interaction [42]. In this regard, we found that the expression of sucrose synthase (Medtr2g044070) was five-fold upregulated in MtN5ox inoculated roots. Besides sugar transporters, the expression of seven transcripts coding for ATP-binding cassette (ABC) transporters, which are proteins that are implicated in the transport of a variety of compounds, such as drug, peptides, organic anion conjugates, fatty acids, and lipids [69], was modulated in MtN5oxinoculated roots (Table 1).

#### 3.3.3. Genes Related to Metabolic Changes Relevant for the Early Response to Rhizobia

Several DEGs with a putative role in the host responses to rhizobia were detected in the comparison MtN5ox vs. WT (Table 2 and Supplementary Table S2).

One of the up-regulated DEGs (*Medtr3g106430*), codes for the SPFH/band 7/PHB domain membrane-associated family protein, also known as FLOT4. Plant flotillins are membrane proteins present in lipid rafts that colocalize with the NF receptor LYK3 in microdomains of the root hairs [70]. Flotillins are responsible for membrane shaping during the early symbiotic events, such as IT initiation and elongation, and the final endocytosis of the bacteria into the plant cells [34]. In a previous work, we demonstrated that *FLOT4* expression was strongly reduced in *MtN5*-silenced plants, proving that MtN5 acts upstream of FLOT4 and supporting the idea that MtN5 participates in the events preceding IT development [16]. In addition, in MtN5ox inoculated roots three transcripts (*Medtr8g080180*, *Medtr8g031370*, *Medtr7g091990*), coding for the carboxy-terminal region of remorin, were downregulated (Table 2). Remorins, like FLOT4, are microdomain protein markers, interacting with the symbiosis receptor kinases in *M. truncatula* [71,72]. In MTN5ox roots, PUB1 (*Medtr5g083030*), which is a plant U-box E3 ubiquitin ligase 1, is downregulated after rhizobial infection (Table 2). PUB1 interacts with LYK3 and with the symbiotic receptor kinase DMI2, and represents a negative regulator of rhizobial and arbuscular mycorrhizal symbioses [73]. Two transcripts (*Medtr8g072640*, *Medtr5g024880*) coding for 3-hydroxy-3-methylglutaryl coenzyme A reductase (HMGR), enzyme that is involved in isoprenoid biosynthesis, were modulated in MtN5ox roots (Table 2). A *M. truncatula* isoform of HMGR (HMGR1) was demonstrated to interact with the SYMRK receptor (MtDMI2), and to be required for efficient nodulation [74]. The *Medtr5g024880* transcript, five-fold upregulated in MtN5ox roots, codes for the HMRG3 isoform that was also proved to be able to bind, albeit weakly, the DMI2 receptor [74].

During the early phases of infection, the invasion of root by the rhizobia requires modifications of the plant cell wall, rearrangement of the cytoskeleton and novel production of membranes [75]. Cell wall degrading enzymes implicated in cell wall loosening, might play a role in the remodelling of plant cell walls during root nodule symbiosis [76]. A quite large number of transcripts that are related to cell wall and cytoskeleton modifications were modulated in MtN5ox inoculated roots (Supplementary Table S2). For instance, several transcripts (*Medtr1g0862101*, *Medtr4g0257301*, *Medtr8g023150*, *Medtr8g023310*, *Medtr7g108905*, *Medtr1g0081401*) coding for pectinesterase/pectinesterase inhibitors were found either up or downregulated in comparison with WT inoculated roots. Other transcripts coding for cell wall-related proteins, including different cellulose synthases, expansins, polygalacturonases, xyloglucan-endotransglucosylases, α-1,4-glucan-protein synthases, pectate lyases, xyloglucanase-specific endoglucanase inhibitor protein were downregulated.

The first phases of root nodule symbiosis involve the perturbation of hormonal homeostasis, mainly resulting in modification of auxin transport and induction of cytokinin signalling [77]. Genes that are involved in auxin transport (*Medtr5g024970*, *Medtr8g107360*, *Medtr1g029190*, *Medtr1g023180*, *Medtr1g051120*) were repressed in MtN5ox inoculated plants as compared with WT ones. These findings are in accordance with previously published data regarding the inhibition of auxin transport and the consequent transient accumulation of auxin at the site of nodule primordia formation [77]. Another hormonal change observed in MtN5ox roots concerns ethylene biosynthesis; a transcript (*Medtr8g028600*) coding for 1-aminocyclopropane-1-carboxylate synthase was 9-fold upregulated. Besides changes in the expression of genes related to the initial phases of infection, we detected a five-fold induction of the transcript (*Medtr5g084080*) coding for a nodule-specific glycine rich peptide (GRP) (Table 2) that is putatively involved in nodule maturation [78,79]. Nodule specific GRPs are a well-represented group of secreted peptides that are identified in *M. truncatula* and other closely related legume species [78], which are proposed to act as antimicrobial peptides, and, similarly to NCR, to play a role in the control of bacteroid differentiation [78].

#### 3.3.4. DEGs Related to Lipid Metabolism

In MtN5ox inoculated roots, we identified several DEGs related to lipid metabolism and lipid transport across membranes (Table 3 and Supplementary Table S2).

Regarding lipid biosynthesis, a gene (*Medtr6g086060*) involved in sphingolipid production was downregulated and three genes (*Medtr2g049790*, *Medtr1g082300*, *Medtr4g115920*) related to galactolipid synthesis were modulated (two upregulated and one downregulated). Sphingolipids are common components of membrane lipid rafts and in *M. truncatula* the NF receptor complex seems to localize in lipid rafts at the root hair plasma membrane [80]. In addition, three transcripts (*Medtr8g089180*, *Medtr5g009160*, *Medtr8g096880*) coding for an UDP-d-glucose/UDP-d-galactose 4-epimerase were modulated (Supplementary Table S2). The UDP-d-glucose/UDP-d-galactose 4-epimerase functions in the biosynthesis of d-galactose, a biosynthetic precursor of galactolipids. Galactolipids are quite abundant in plastidial and symbiosome membranes [81].

Interestingly, the majority of DEGs related to lipid metabolism were represented by genes implicated in phospholipid signalling such as a myo-inositol 1-phosphate synthase (*Medtr3g087590*), a phosphatidylinositol-specific phospholipase C (*Medtr5g071010*), a phosphatidylinositol 3,4- and 4,5 kinases (*Medtr0009s0120, Medtr1g062740*, *Medtr7g017360*, *Medtr5g009120*), a polyphosphoinositide-binding protein (*Medtr3g098530*), and a sec14p-like phosphatidylinositol transfer family protein (*Medtr2g031610*). Sec14domain proteins play a role in many cellular activities [82], including root hair biogenesis [83] and membrane-trafficking [84]. In this regard, the expression of a transcript (*Medtr1g068945*) coding for a Sec24 plant–like protein, a crucial component of the vesicular transport of proteins and lipids, was 24-fold increased in MtN5ox inoculated roots. All of the transcripts that were related to phosphoinositol metabolism were downregulated in MtN5ox inoculated roots, except for one (*Medtr0009s0120*). These data suggest that the phosphatidylinositol signalling pathway is hampered in MtN5ox roots. 

Another interesting transcriptional change that was observed in MtN5ox roots was the downregulation of two genes (*Medtr8g018420, Medtr8g018730*) that were involved in oxylipin synthesis. Oxylipins, which include jasmonic acid, were shown to inhibit the host response to rhizobia at an early phase of infection [85].

### 3.4. Lipid Profiles of Rhizobia-inoculated MtN5ox and WT Roots

Transcriptomic changes related to lipid metabolism and signalling observed in MtN5ox roots challenged with rhizobia might be linked to the capacity of MtN5 to bind and transfer lipids. To verify whether these transcriptomic changes are associated with alterations in the lipid composition of the MtN5ox roots, we analysed the lipid profile of MtN5 and WT roots of *M. truncatula* 72 h after rhizobial inoculation.

**Figure 4:**
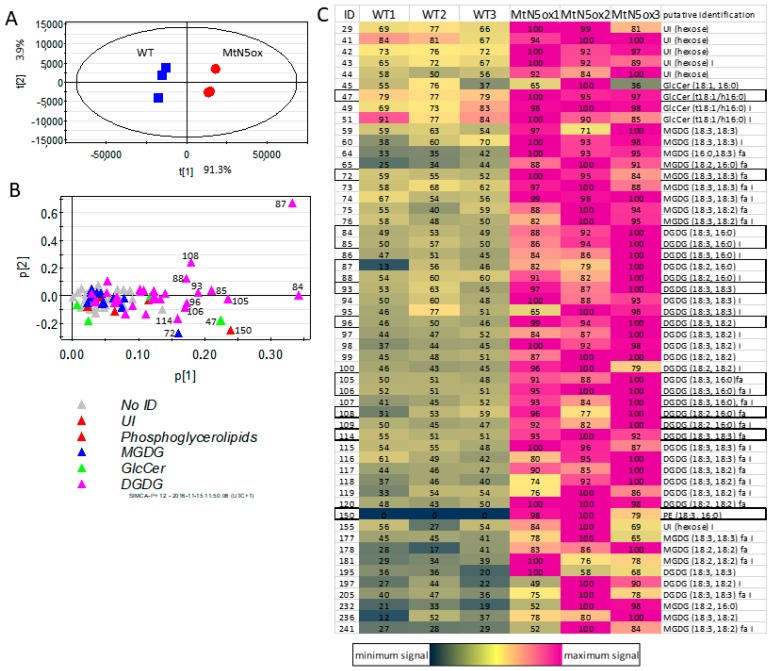
Principal Component Analysis (PCA) score plot (**A**) and loading plot (**B**) of WT and MtN5ox roots. (**C**) heat map (individual row-based) of signal intensity (in arbitrary units) of annotated lipid (*m/z* features). WT = roots from wild type control plants; MtN5ox = roots from *MtN5* overexpressing plants. The lipids labelled in the PCA loading plots are outlined in the heat map. UI, unidentified hexose-containing lipids; MGDG, Monogalactosyldiacylglycerol; GlcCer, Glucosyl Ceramide; DGDG, Digalactosyldiacyl glycerol.

The lipidic fraction of three independent pools of roots from WT and MtN5ox plants were analysed by HPLC-APCI-MS, with a method that was optimized for lipid analysis, allowing the detection of 97 main *m/z* features, 54 of which were putatively annotated. They included MGDG, MGDG, two GlcCer, one PE containing phosphoglycerolipid and some other hexose-containing unidentified lipids, plus their fragments, adducts, and isotopes (Supplementary Table S3). The raw chromatographic analysis showed consistent differences between the three pools of WT and MtN5ox roots (Supplementary Figure S3). Several lipids, including some DGDGs, one MGDG, the PE phospholipid, and the two GlcCer showed higher signals in MtN5ox roots when compared with WT ones. The data matrix obtained with MZ mine software was imported in Simca plus (Umetrics) for the multivariate analysis. The PCA showed a clear separation of the three samples from WT from the three samples from MtN5ox plants (Figure 4A).

The accumulation of higher levels of lipids in transformed plants is also shown in the heat maps, which show the relative abundance of annotated (Figure 4C) and not annotated lipids (Supplementary Figure S4). The loading plot showed that almost all of the lipids positively characterized the roots of MtN5ox plants, with a stronger contribution of some DGDG, one MGDG, one GlcCer, and the PE phosphoglycerolipid (Figure 4B). All of the lipids, except the phosphoglycerolipid, were detected both in WT and MtN5ox roots, while the PE-based (18:3, 16:0) phosphoglycerolipid was barely detectable in WT plants.

## 4. Discussion

The plant nsLTPs represent a very large family of secreted proteins that are characterized by a conserved cysteine pattern and specific structural features that allow for the interaction with a variety of lipidic molecules. Their lipid binding capacity seems to be exploited in a plethora of physiological processes, such as abiotic and biotic stress responses, cutin formation, pollination, and symbiosis. The involvement of LTPs in plant symbiotic interaction with nitrogen-fixing bacteria is restricted to two examples: a type I LTP of *A. sinicus* (AsE246) and the *M. truncatula* N5 a LTP with intermediate features between type I and II LTPs. Both AsE246 and MtN5 are required for efficient rhizobial infection and nodulation, but displayed different patterns of expression during symbiotic interaction [16,17]. When considering the general antimicrobial activity of LTPs and their lipid binding ability, these proteins could interact with rhizobia membranes and interfere with the bacterial infection and differentiation. On the other hand, they could participate in the synthesis and/or the rearrangements of membranes occurring during perception of bacteria and the formation of infection thread and symbiosome. 

The approach we used to further unravel the function of MtN5 consisted in the analysis of the transcriptomic changes that were caused by the downregulation and overexpression of the gene in the roots after rhizobial inoculation. We focused the analysis on early stages of infection (three dpi), because MtN5 is induced starting from the pre-infection stage, and our previous data revealed that it is implicated in controlling rhizobia spread into the roots. At three dpi, MtN5hp roots show an enhanced responsiveness to rhizobia (increased number of curling events), whereas the MtN5ox and WT inoculated roots exhibit a similar response to *S. meliloti*. At successive stages of infection, MtN5hp roots maintain an increased sensitivity to bacteria, but the invasion of nodule primordia and the production of mature nodules are impaired. On the contrary, the overexpression of MtN5 favours at a later stage (seven dpi) the infection and successively also the nodule invasion. The transcriptomic analysis of MtN5hp plants demonstrated limited changes in gene expression at the level of the entire root. One of the possible explanations could be found in the expression pattern of *MtN5*, which is locally induced at the sites of rhizobia attachment in the first phase of infection. The principal transcriptomic modifications that were observed in MtN5hp roots concern genes that are related to membrane transport and response to biotic stress. Indeed, the initial reaction of the host to the symbiont includes membrane protein rearrangements and the induction of defence genes. The immune response is successively weakened probably by the coordinated activities of the plant and the bacteria [80,86]. However, the mechanisms that facilitate the host receptivity are still unclarified. The changes that are observed in MtN5hp plants are in accordance with a role of MtN5 in limiting the uncontrolled spreading of the bacteria in the roots. This response appeared specific for the rhizobial symbiosis, since *MtN5* expression is not induced by mycorrhiza infection and MtN5hp roots are efficiently colonized by *R. irregularis*. Notably, the downregulation of *MtN5* resulted in the induction of two genes coding for NCR peptides. NCRs possess anti-microbial activity and act in the nodules regulating bacteroid differentiation [30]. Inside bacteroids, NCRs can affect different targets such as cell cycle-related proteins and stress response factors [30]. Recently, it has been demonstrated that NCRs are temporally regulated during nodule formation to facilitate the symbiosis [87]. The unregulated expression of NCRs in MtN5hp roots at three dpi could provoke an altered defence response. Overall, the downregulation of MtN5 seems to result in the augmentation of defence response mounted in the roots against rhizobia possibly as a consequence of the increased bacteria spreading. The persistence of the defence response eventually leads to reduce nodulation capacity.

The overexpression of MtN5 results in a large reprogramming of the root transcriptome, although at three dpi the response to rhizobia inoculation was apparently similar to that of WT. The observed transcriptome modifications may derive from the constitutive expression of the gene and/or from a different response of MtN5ox roots to the symbiont inoculation. In any case, the differential transcriptomic responses of MtN5ox roots to rhizobia at this stage of infection can be predictive of the successive phenotypic outcomes (i.e., increased number of root curling events, invaded primordia and nodules). The root transcriptome reprogramming mainly concerns the modification of regulatory pathways, since genes coding for different classes of TFs account for one of the major DEGs categories. None of the TFs that were modulated by the overexpression of MtN5 has already been documented as directly implicated in the rhizobial symbiosis, therefore these TFs might represent a dataset of new candidate genes putatively involved in the regulation of the legume-rhizobia symbiosis.

The picture that emerges from the transcriptomic survey is congruent with the potentiation of mechanisms that favour the successful colonization of the roots by the rhizobia. A low concentration of nitrogen in the soil is a prerequisite for the establishment of a symbiotic interaction between legume and rhizobia. This condition is possibly reflected in the damping of molecular mechanisms deputed to nitrogen uptake and distribution. It is interesting to observe that in MtN5ox inoculated roots, many genes coding for transporters of nitrate and organic form of nitrogen (peptides and amino acids) are downregulated. It is possible that among other mechanisms, MtN5ox plants display a higher responsiveness to rhizobia than WT, also by directly and indirectly affecting the uptake of NO_3_^−^ and the transport of nitrogen assimilation products. 

The perception of NFs by host receptor complexes triggers the signalling cascade that leads to root hair deformation and nodule primordia invasion. In MtN5ox roots, we observed the modulation of genes coding for membrane proteins (i.e., FLOT4, remorin, PUB1, HMGR) that interact with NF receptor LYK3 and/or with the symbiosis receptor kinase DMI2. These proteins are thought to participate in the regulation of the activity of the receptor complexes, and their misfunction can affect early phases of infection such as root hairs curling and ITs formation [34,73]. The upregulation of FLOT4 that interact with the actin cytoskeleton facilitating membrane rearrangements [34], and the downregulation of PUB1 an E3 Ubiquitin ligase, playing an inhibitory role in ITs formation and nodule primordia infection [73], might favour the rhizobia recognition and entry in MtN5ox roots. Furthermore, in MtN5ox roots the broad modification of genes coding for proteins that are involved in the synthesis and the assembly of cell wall components and cytoskeleton rearrangement might be indicative of an active process of cell wall reorganization.

The formation of the ITs, as well as the release of rhizobia in the nodule cells, requires the synthesis of new membranes. Modifications of the lipidic composition and the integration of new transporters occur in the symbiosome membrane [81]. Therefore, the progression of rhizobia infection requires an active synthesis and intracellular trafficking of lipids. LTPs could be involved in lipid trafficking targeting infection threads and symbiosome membranes, as suggested for the AsE246 LTP. AsE246 is able to bind DGDG, a major constituent of the symbiosome membrane [81,88] and colocalize with the symbiosome [17]. Silencing of *AsE246* caused a significant reduction in the abundance of lipids, including DGDG, in the nodules [17]. In our study, we observed that the overexpression of N5 determined an increased content of lipids at the level of the entire root. The lipids that mostly contribute to discriminate the profile of MtN5ox from that of WT roots, are a GlcCer, a PE, a MGDG, and several types of DGDG. The MGDG and DGDG are nonphosphorus galactoglycerolipids that are highly abundant in photosynthetic organelles [89], but they are also present in plasma membrane and symbiosome membrane [81,88]. Notably, during phosphorus (P) starvation, the phospholipids are degraded to release the phosphate needed for cellular processes, and concomitantly galactoglycerolipids are exported to extraplastidial membranes to replace phospholipids [90,91]. Phosphate availability is a major constraint for the symbiotic nitrogen-fixation, a highly energy-demanding metabolic process [92]. Consequently, the presence of the nonphosphorus MGDG and DGDG in the symbiosome membrane might be explained as a metabolic strategy that is mounted by the plant to save phosphate [81]. In MtN5ox roots, the increased amount of MGDG and DGDG is linked to the increased expression of genes that are implicated in the biosynthesis of galactolipids and d-galactose, their biosynthetic precursor.

Besides being component of membranes, lipids also serve as substrates for the generation of numerous signalling lipids, such as phosphatidic acid, phosphoinositides, sphingolipids, lysophospholipids, and oxylipins [93]. These signalling molecules are usually generated by modifying enzymes, like phospholipases. In this regard, the overexpression of MtN5ox determines the downregulation of several genes that are implicated in the phosphatidyl inositol signalling pathway, as well as phosphatidyl inositol-specific phospholipase C (PI-PLC). Phospholipase and phospholipid-derived molecules have a central role during early defence signalling events [94]. Rhizobia are initially perceived as intruders by the host plant and the subsequent establishment of a successful symbiosis may depend on escaping or suppressing the defence response. It has been demonstrated in *Medicago sativa* cell suspension that the PI-PLC pathway is induced by Nod factors and fungal elicitors, whereas phospholipase D (PLD) only by NFs [95]. The inhibition of PI-PLC activity blocks the early defence-activated responses, such as medium alkalinisation, thus placing PI-PLC at the centre of plant innate immunity [94]. The activation of PI-PLC by Nod factor may represent an initial defence signalling, on the contrary, PLD activation seems to attenuate or modify the defence reaction [95]. In this regard, we previously demonstrated that MtN5 functioning is dependent on phospholipase D activity [16]. 

Overall, our findings demonstrate that MtN5 is involved in modifying the root lipid profile, favouring the early synthesis of galactoglycerolipids, which are known to be targeted to the symbiosome. Although the lipidic composition of ITs membranes has not been investigated, we cannot exclude that these membranes, from which the symbiosome derived, could be subject to a similar lipid rearrangement. Thus, during the early phase of infection, MtN5 would be implicated in the production and remodelling of membranes that are required for rhizobia invasion. Besides these effects, MtN5 activity would facilitate the symbiont to overcome the plant immune response by acting on the phospholipid signalling pathway. This hypothesis is in accordance with the existence of an interplay between PI-PLC and PLD signalling pathways crucial to discriminate between defence and symbiotic responses [94,95].

## Figures and Tables

**Table 1:** Differentially expressed transcripts involved in transport metabolism discussed in the text.

Genome ID	Description	Fold Change (MtN5ox vs. WT)
Medtr4g098800	peptide transporter	−10.74
Medtr3g100980	peptide/nitrate transporter	−7.32
Medtr2g094270	major intrinsic protein (MIP) family transporter	−6.91
Medtr8g089340	cationic amino acid transporter	−5.82
Medtr8g085630	neutral amino acid transporter	−5.47
Medtr8g087710	MIP family transporter	−5.41
Medtr4g114340	peptide/nitrate transporter	−5.1
Medtr3g070210	MIP family transporter	−5.04
Medtr4g099260	high-affinity potassium transporter	−4.99
Medtr4g104510	transmembrane amino acid transporter family protein	−4.88
Medtr4g134730	chloride channel (ClC1) protein	−4.76
Medtr3g087740	sulphate/bicarbonate/oxalate exchanger and transporter sat-1	−4.59
Medtr3g082700	high affinity inorganic phosphate transporter	−4.49
Medtr1g041695	phosphate transporter PHO1-like protein	−4.4
Medtr5g038060	peptide/nitrate transporter	−4.37
Medtr1g071530	sulphate/bicarbonate/oxalate exchanger and transporter sat-1	−4.11
Medtr6g016275	transmembrane amino acid transporter family protein	−4.03
Medtr1g006490	MIP family transporter	−3.47
Medtr8g061090	oligopeptide transporter (OPT) family protein	−3.47
Medtr2g012470	transmembrane amino acid transporter family protein	−3.44
Medtr1g109380	amino-acid permease BAT1-like protein	−3.43
Medtr2g101090	drug resistance transporter-like ABC domain protein	−3.1
Medtr4g069500	polyol/monosaccharide transporter 1	−3.02
Medtr0294s0020	proton-dependent oligopeptide transport family protein	−2.9
Medtr3g108320	potassium channel KAT3 protein	−2.81
Medtr4g083570	ZIP zinc/iron transport family protein	−2.7
Medtr1g098240	nucleotide/sugar transporter family protein	−2.67
Medtr5g075960	transporter family ABC domain protein	−2.55
Medtr4g011600	sulphate transporter-like protein	−2.39
Medtr1g075640	phosphate transporter PHO1-like protein	−2.35
Medtr7g017630	transmembrane amino acid transporter family protein	−2.18
Medtr4g123990	ABC transporter B family protein	−2.14
Medtr6g008690	peptide/nitrate transporter plant-like protein	−1.94
Medtr6g065650	ClC1 protein	−1.88
Medtr7g088820	nitrate transporter 1	−1.78
Medtr2g020710	sugar porter (SP) family MFS transporter	2.33
Medtr2g028770	magnesium transporter CorA family protein	2.33
Medtr8g088200	high-affinity potassium transporter	2.76
Medtr5g068580	vacuolar iron transporter-like protein	3.42
Medtr4g069430	nucleotide-diphospho-sugar transferase family protein	3.52
Medtr1g104780	SP family MFS transporter	3.75

**Table 2:** Differentially expressed transcripts known to be involved in the nodulation process and discussed in the text.

Genome ID	Description	Fold Change (MtN5ox vs. WT)
Medtr7g091990	Carboxy-terminal region remorin	−4.57
Medtr8g080180	Carboxy-terminal region remorin	−4.54
Medtr8g031370	Carboxy-terminal region remorin	−1.94
Medtr5g083030	Ubiquitin-protein ligase, PUB17	−1.93
Medtr5g084080	Nodule-specific Glycine Rich Peptide	5.45
Medtr3g106430	Flotillin-like 4	3.45
Medtr5g024880	3-hydroxy-3-methylglutaryl-coenzyme A reductase-like protein	5.05

**Table 3:** DEGs involved in lipid metabolism.

Genome ID	Description	Fold Change (MtN5ox vs. WT)
Medtr4g087830	phospholipase A1	−6.41
Medtr3g087590	myo-inositol 1-phosphate synthase	−6.32
Medtr8g018420	seed linoleate 9S-lipoxygenase	−5.48
Medtr4g053785	gland-specific fatty acyl-CoA reductase	−4.97
Medtr7g076900	calcium-dependent lipid-binding (CaLB domain) family protein	−4.01
Medtr8g018730	seed linoleate 9S-lipoxygenase	−3.91
Medtr5g071010	phosphatidylinositol-specific phospholipase C	−3.49
Medtr4g107850	(CaLB domain) family protein	−3.49
Medtr6g086060	serinc-domain serine and sphingolipid biosynthesis protein	−3.16
Medtr7g104220	GDSL-like lipase/acylhydrolase	−3.15
Medtr2g031610	Sec14p-like phosphatidylinositol transfer family protein	−3.06
Medtr5g094230	glycerophosphoryl diester phosphodiesterase family protein	−2.95
Medtr5g009120	phosphatidylinositol 3-and 4-kinase family protein	−2.86
Medtr8g070040	lipid transfer protein	−2.86
Medtr1g106875	lipase	−2.45
Medtr3g079340	enhanced disease susceptibility protein	−2.39
Medtr3g098530	polyphosphoinositide-binding protein	−2.22
Medtr7g017360	phosphatidylinositol 3-and 4-kinase	−2.22
Medtr4g115920	monogalactosyldiacylglycerol synthase	−2.19
Medtr1g062740	phosphatidylinositol-4-phosphate 5-kinase family protein	−2.16
Medtr7g113660	mevalonate kinase	−1.81
Medtr6g006910	phospholipid-transporting ATPase-like protein	−1.81
Medtr7g117475	phospholipid methyltransferase	−1.63
Medtr1g097850	choline/ethanolamine kinase	1.49
Medtr1g041495	glycolipid transfer protein (GLTP) family protein	1.62
Medtr2g020020	alpha/beta-hydrolase superfamily protein	1.70
Medtr5g012880	phosphatidylserine decarboxylase	1.86
Medtr8g031400	GDSL-like lipase/acylhydrolase	2.23
Medtr0009s0120	phosphatidylinositol 3-and 4-kinase	2.26
Medtr1g070195	alpha/beta-hydrolase superfamily protein	2.87
Medtr1g082300	breast carcinoma amplified sequence 3 protein	3.02
Medtr2g049790	CBL-interacting kinase	3.21
Medtr7g109830	long-chain fatty acyl CoA ligase	4.12
Medtr7g083130	esterase/lipase/thioesterase family protein	4.37
Medtr7g090470	triacylglycerol lipase SDP1	5.41
Medtr1g068945	protein transporter Sec24-plant-like protein	23.83

Genome ID, transcript description, fold change value.

## Supplementary Materials

The following are available online at www.mdpi.com/2073-4425/8/12/396/s1. Figure S1: (A) Principal fungal structures observed in WT, *MtN5hp* and *MtN5ox* roots during the infection with *R. irregularis*. Figure S2. Validation of a set of DEGs by qRT-PCR analysis. Figure S3: Raw HPLC-APCI-MS chromatograms of roots of WT (black) and *MtN5ox* roots (red). Figure S4: Heat map (individual row-based) of signal intensity (in arbitrary units) of not annotated lipid (*m*/*z* features) in WT plants and *MtN5ox* roots. Table S1: List of DEGs identified by microarray analysis in the comparison MtN5hp-inoculated roots versus WT-inoculated ones. Table S2: List of DEGs identified by microarray analysis in the comparison MtN5ox-inoculated roots versus WT-inoculated ones. Table S3: Identification n. (id), *m*/*z* (negative ionization mode), retention time (rt, in minutes), putative identification, lipid class, P(1) and P(2) loadings, peak area (in arbitrary units) in WT and MtN5ox samples. Table S4: Transcript ID, gene product and list of primers adopted for qRT-PCR analyses.
